# Exploring the experiences of nurses’ moral distress in long-term care of older adults: a phenomenological study

**DOI:** 10.1186/s12912-021-00675-3

**Published:** 2021-08-31

**Authors:** Alireza Nikbakht Nasrabadi, Ahmad Hasyim Wibisono, Kelly-Ann Allen, Ameneh Yaghoobzadeh, Yee Bit-Lian

**Affiliations:** 1 School of Nursing and Midwifery, Tehran University of Medical Sciences, Tehran, Iran; 2 Medical Surgical Nursing Department, School of Nursing, Brawijaya University, Malang, Indonesia; 3 School of Educational Psychology and Counseling, Faculty of Education, Monash University, Clayton, Australia; 4 Centre for Wellbeing Science, University of Melbourne, Melbourne, Australia; 5 School of Nursing, Arak University of Medical Sciences, Arak, Iran; 6 Nursing Division, School of Health Sciences, International Medical University, Kuala Lumpur, Malaysia

**Keywords:** Moral distress, Older adult, Aging, Nursing, Qualitative research, Intensive care, Critical care

## Abstract

**Background:**

Moral distress is a poorly defined and frequently misunderstood phenomenon, and little is known about its triggering factors during ICU end-of-life decisions for nurses in Iran. This study aimed to explore the experiences of nurses’ moral distress in the long-term care of older adults via a phenomenological study.

**Methods:**

A qualitative, phenomenological study was conducted with 9 participants using in-depth semi-structured interviews. The purpose was to gain insight into the lived experiences and perceptions of moral distress among ICU nurses in hospitals affiliated with Tehran University of Medical Sciences during their long-term care of older adults.

**Results:**

Five major themes are identified from the interviews: advocating, defense mechanisms, burden of care, relationships, and organizational issues. In addition, several subthemes emerged including respectful end of life care, symptom management, coping, spirituality, futile care, emotional work, powerlessness, relationships between patients and families, relationships with healthcare teams, relationships with institutions, inadequate staffing, inadequate training, preparedness, education/mentoring, workload, and support.

**Conclusions:**

This qualitative study contributes to the limited knowledge and understanding of the challenges nurses face in the ICU. It also offers possible implications for implementing supportive interventions.

## Background

Nursing, as a profession, requires regular ethical decision making, which is a vital component of the nurse-patient relationship [1]. In other words, nurses make decisions based on their moral beliefs during the different stages of a patients’ life cycle [2]. These decisions can be influenced by the unique situation or context a patient is in, a patient’s family or the perspectives of other physicians and nurses [3, 4].

Moral distress is one of the most challenging ethical issues in nursing [5]. Jameton [6] defined moral distress as a psychological discomfort that occurs when a person is unable to complete what they believe to be an ethically responsible action due to internal or external factors. Based on this definition, nurses may have professional work requirements that conflict with their personal morals and beliefs [5].

Corley [7] drew from Jameton’s work in her description of the concept of moral distress. She defined it as the physical and intellectual pain accompanied by worrying about the impact of decisions involved in patient care on interpersonal relationships [4].

Moral distress has undesirable consequences on nurses’ wellbeing [8], potential job loss, diminished patient care, and general health outcomes [5, 9]. Specific problems include insomnia, heart arrhythmias and palpitations, anxiety, feelings of unimportance, and depression. Therefore, situations perceived as morally distressing predispose nurses to physical and psychological health problems [10].

Many studies have investigated the moral distress of nurses in intensive care units (ICUs). Major contributors to moral distress include providing end-of-life care, decision making in organ and blood donation [9], patient pain management [8, 9, 11], lack of organizational support, resources, feelings of incompetence or inexperience [12, 13], and conflicting values in care between other medical professionals [11]. Also, the time involved in patient care has a significant effect on the severity of moral distress [12]. Nurses are constantly challenged with the reality of patient suffering [8], even though active treatment and end-of-life decisions are made by the physicians. Nurses are involved in the care of critically ill and dying patients, their mandate is to provide patient and family-centered care in intimate and often intense situations such as in the intensive care unit (ICU) [14]. In this heightened emotional climate, ethical decisions have to be made that may be contested or divisive within the multidisciplinary healthcare team [15]. Research reviews indicate that end-of-life care in particular has long been recognized as engendering moral distress due to the situations inherent in caring for a dying person [16–19].

Compassion fatigue is commonly found in staff caring for patients and their families faced with critical illness and death [20]. Peer supervision has been found to be effective for reducing the moral distress of ICU nurses, that is where nurses discuss ethical issues [21] with their peers, experienced educators, or clinicians [11]. Other strategies are widely used to improve decision-making in end-of-life care, including multidisciplinary team meetings and providing additional support to specialist care providers and end-of-life decision-makers [22]. Such strategies are  believed to enable ICU nurses to confront moral distress and at the same time, enjoy the challenges of working in an ICU environment [11]. Distress may also be experienced when the expectations of patient family members are unrealistic or not in the patient’s best interests (i.e., when a patient’s safety is compromised) [23], or when the medical team overrides the patient’s or family’s wishes [21]. Furthermore, the ethical sensitivity of the carer appears to be proportionate to the distress experienced [16].

However, little is known about moral distress in the care of older adults. Due to the physical health problems older adults may experience and their dependence on others in the different aspects of their life, a special emphasis on ethical issues in the care of all older patients is essential [24, 25]. In addition, nurses often encounter patients who spend the final moments of their lives asking challenging questions about the intensive care they are receiving [26]. In contrast, the existence of a long-term relationship with patients has been cited as a factor in reducing moral distress [11, 27].

Alvita Nathaniel’s Theory of Moral Reckoning in Nursing is a logical, systematic, and explanatory theory of moral distress and its consequences [28]. The first stage is the stage of ease which has four conditions (*becoming, professionalizing, institutionalizing, and working*). In the *becoming* condition, nurses’ core beliefs are evident from their experiences in caring for others, their commitment to upholding professional and institutional norms, and their experiences when core values are challenged. *Professionalizing* condition involves adhering to professional and ethical standards while *institutionalizing* condition involves the work environment and culture which can be congruent or not with nurses’ core beliefs and professional norms. Lastly, the *working* condition notes that the type of work varies for each nurse with challenges and great rewards. These conditions are incorporated in the act of patient care. Nevertheless, a morally troubling event may challenge the integration of core beliefs with professional and institutional norms, resulting in “situational binds” that create a juncture in nurses’ lives. Binds involve serious conflicts within individuals resulting in moral ethical dilemmas. Nurses are caught in these situational binds and often experience a sense of helplessness. Unresolved situational binds result in negative personal and patient care consequences of moral distress. The subsequent stage of resolution results in a choice of either making a stand (confronting the situational binds) or giving up. The final stage of reflection allows nurses to reflect on their behaviors and actions which may last a lifetime and include remembering, telling the story, examining conflicts, and living with consequences [29].

Although many studies have assessed moral distress among ICU nurses, there are potentially large differences across cultural and contextual factors, which play important roles in ethical dilemmas and moral distress [28, 30–32]. These studies found that nurses from diverse societal and cultural backgrounds have dissimilar ethical and religious understandings, which impact their moral distress. This evidence suggests that generalisations between cultures should be treated with caution.

Most studies on moral distress and nurses have been conducted in Western landscapes. To date, little is known about Islamic perspectives. Shorideh et al. emphasized that moral stressors should be contextualized in cultural perspectives [28]. Therefore, this study presents Iran as a country with an Islamic culture and a healthcare system that is unique from other countries [28, 33]. Shahriari et al. indicated that the Iranian healthcare system is dominated by pillars underpinned by religious and cultural views [29]. In Iran, patient care standards are directed by Iranian beliefs in Islamic moral and ethical [32]. Thus, nurses from different social and cultural backgrounds have different ethical and religious knowledge which may influence their moral distress [30].

Overall, consideration of moral distress in various cultural contexts and populations is necessary for improving the understanding of moral distress in nursing [34].

### Aim

This study aimed to explore the nurses’ experience of moral distress in the long-term care of older adults via a phenomenological study.

## Methods

### Study design & setting

This qualitative study followed the phenomenological method described by Van Manen [35]. Its goal was to explore the nurses’ experience of moral distress in the long-term care of older adults. According to Van Menen, interpretive phenomenology offers a systematic approach that allows a phenomenon to be analyzed, interpreted, and explored, in order to gain a deeper understanding of lived experiences via the interpretation process.

### Procedure and participants

Participants of this research were nurses who work in the ICUs of hospitals affiliated with Tehran University of Medical Sciences. The inclusion criteria involved having a Bachelor’s or Master’s degree and working in an ICU for at least a year. This project was approved by the ethical committee of Tehran University of Medical Sciences.

A purposive sampling method is common in phenomenological studies. Therefore, this study used this type of sampling method with the intention of obtaining in-depth information from each individual. In purposive sampling, participants are selected based on their specific characteristics. According to Van Manen’s analysis, stop-sampling (i.e., data saturation) includes abstractness, richness, relatedness, and depth. In this study, 9 ICU nurses were included: 4 males and 5 females.

### Data collection

In-depth and semi-structured interviews were used to collect information. Interview questions were asked based on the purpose of the study. Three of these questions included: 1. Please share your first care experience in the clinical setting; 2. How do you feel when you are taking care of an older person who has been hospitalized for a long time in the ICU?; 3. What does caring for an older person look like in your point of view? Additional research questions were asked based on the information the patients provided in order to achieve an in-depth interview including “Can you explain more to me?“ and “Can you give me an example?” Interviews were conducted by one of the researchers (AY) with training in interview procedures and each interview was checked by ANNA. More interviews were conducted as needed. In order to assure the validity and accuracy of the data collection, transcriptions and the coding process were done and checked by ANNA.

On average, each interview lasted between 20 and 60 min. All the interviews were recorded and transcribed verbatim as soon as possible after the interview took place. All participants were coded according to the order of the interview. Van Manen’s 6-step analysis method was used for data analysis. These steps are as follows: turning to the nature of lived experience, investigating experience as we live it, reflecting on the essential themes that characterize the phenomenon, describing the phenomenon in the art of writing and rewriting, maintaining a strong and orientated relation to the phenomenon, and balancing the research context by considering both the parts and the whole [35].

Two holistic and selective approaches of Van Manen’s thematic analysis process were used to extract themes. According to this approach, the interview was transcribed on paper, then reviewed several times to gain an overall understanding. Afterward, it was written in 2–3 paragraphs, which helped the researcher immerse in the data. Then based on a selective approach, the text of each interview was read out loud, and the sentences or phrases that seemed to describe the essence of the moral distress in long-term care of older adults were selected and revealed. Research team members then shared and discussed the topics and extracted themes.

This process kept motivating other interviews. During these conversations, new findings were obtained, and changes were made to previous findings. The process of returning to the texts was repeated multiple times, in order to resolve any disagreements and inconsistencies in the interpretations. This process continued until the themes were best communicated with each other.

### The trustworthiness of the study

Lincoln and Guba’s method was also used in the present study [36]. The assessed items of this method were as follows: credibility, dependability, transferability, and confirmability. To examine the validity of the research, a trusted relationship was established with the participants. Each interview was provided to the participants after analyzing it, and their comments were sought to confirm the data. Also, quotes reported were recorded verbatim. This study was conducted in stages with the participation of several reviewers from the research team. Moreover, the reviewers’ suggestions were used throughout the research process. An external audit was used to assess the trustworthiness of the study [34]. Ultimately, the audit process attested to the dependability of the study from a methodological standpoint, and the confirmability of the study by reviewing the data, analysis and interpretations, and assessing whether or not the findings accurately represent the data. In essence, the audit examines both the process and product of the inquiry to determine its trustworthiness. In this study, other advisors/supervisors and evaluators who were experts in qualitative research assess each stage of the research and provided suggestions as needed [37].

One of the key issues in qualitative research is the role of the researcher in eliciting data. The researcher as an instrument offers opportunity to understand and explore an individual’s experiences and perceptions of the phenomena in question. In order to appropriately conduct qualitative research, the researcher should have the necessary experience and skills, and the ability to communicate [36].

## Results

Participants were chosen through the use of purposive sampling. The study included five female and four male Critical Care Nurses (CCNs). All of the study participants have earned their Bachelor of Science in Nursing (BSN). Table 1 indicates the demographic information of the participants.

**Table 1:** Characteristics of the participants

Characteristic	N (%) or Mean (SD)
Gender	
* Male*	4 (45 %)
* Female*	5 (55 %)
Age	33.82 (8.06)
Marital Status	
* Single*	1 (11.1 %)
* Married*	6 (66.7 %)
* Widowed*	2 (22.2 %)
Education	
* BSc*	6 (66.7 %)
* MSc*	2 (22.2 %)
* PhD*	1 (11.1 %)
Job Experience (years)	10.35 (7.95)
Income	3,798,000 (654,620)
Shifts	
* Morning Fix*	4 (45 %)
* Evening Fix*	1 (10 %)
* Night Fix*	4 (45 %)

### Identified themes

Five major themes and thirteen subthemes emerged from the lived experiences of the participants (Table 2).

**Table 2:** Major themes and subthemes of nurses` moral distress in long-term care of older adults

Major themes	Sub-themes
Advocating	Good dying Symptom management
Defense mechanisms	Coping Spirituality
Care burden	Futile care Emotional work Powerlessness
Relationship	Relationship between patient and family Relationship with healthcare team Relationship with institution
Organizational issues	Inadequate staffing Inadequate training, preparation, education, or mentoring Workload and Support

### Advocating

Advocating was found to be a component of end-of-life care that could enable nurses to “do the right thing” by attaining the related wishes of older adults. Actually, advocating on behalf of older adults as their desires were identified helped nurses to provide good end-of-life care. Despite these positive features, nurses could also be fearful of the consequences of advocating for their older-adult patients, due to the possibility of losing their jobs or having a complaint made against them if they made a “wrong” decision.

For instance, participant No. 7 stated:

*“I am working in ICU for more than 7 years. I have to be responsible for all the things happened to the patient/s who I care. You know, sometimes I feared about results of this caring. I mean that I am fear about some punishments like losing the income, although I know the responsibility of nurses which we have learnt in during university education!”*

The concept of “respectful end of life care” intricately facilitates “doing the right thing.” The day-to-day practice of end-of-life care among CCNs ensures that the choices of older adults are respected. This goal is achieved by advocating on the patients’ behalf. Almost all of the participants valued knowing the wishes of their older-adult patients, especially as they related to “respectful end of life care”.

CCNs described how they were able to advocate “doing the right thing.” In other words, knowing these things did not always mean that patients achieved their wishe.s Furthermore, when nurses were unable to advocate on behalf of the residents or their relatives, the staff felt powerless to change or influence the care provided, which contributed to their experience of moral distress. Regarding this, participant No. 9 stated:

*“I myself feel more responsibility when I care for end-stage patients like cancer patients in ICU. I know that they eventually will die, but I do my best to do all the needed care. I do believe that, although he/she can’t understand what I am doing, I know that I have to be responsible for his/her care”.*

Major contributors to moral distress include deficits in knowledge related to end-of-life care and symptom management. The interviewed CCNs recognized providing education as one way to improve end-of-life care and symptom management. The following statements were mentioned by participant No. 5:

*“As it is obvious when a patient is admitted to the ICU, he/she needs critical care which requires a competent and knowledgeable nurse to provide all the care as soon as possible. Unfortunately, some nurses can’t control the condition and that makes them feel distressed. Imagine you have a dehydrated old patient who has hypotension. Surely, if you cannot manage this condition, his/her level of consciousness will be decreased soon and make him/her to experience hazardous conditions. Knowledgeable nurses can intervene immediately before negative things happen. It is great that working in such difficult ward-needed certification. This made nurses to be updated and refresh their information. Having experienced nurses in each shift make me feel at ease and confident…”*

### Defense mechanisms

Two of the subthemes indicated that nurses use coping strategies when facing moral distress. Based on the interviews, these methods help nurses control their conditions and more comfortably partake their duties.

When participants were questioned about coping techniques, each of the nine participants stated at least one method for coping with moral distress. All these methods were related to maintaining self-care and managing personal stress. Methods reported by various participants included talking to peers and crying.

Participant No. 2’s beliefs about her colleagues’ coping mechanism were particularly interesting. She stated, *“Talking with everybody else that went through it with you…because nobody else could really understand it the same way*.”

Participant No. 3 said, *“Working as a nurse, especially with older adults who are at the end-stage of life, makes you feel uncomfortable. It will be exacerbated when you continually work with these kinds of patients. So using techniques which distract you to think about these things will help you to adapt more. I myself start doing yoga recently. Participating in this class makes me feel at ease and relaxed. I recommend you to participate, too.*“

More than half of the CCNs in the current study verbalized that they considered themselves to be “spiritual or religious” persons. They stated that spirituality is one of the components of moral distress. For example, participant No. 8 stated that:

*“Moral distress is kind of like an everyday thing particularly in ICU. It’s emotional, spiritual, and even physical at times.”*

Moreover, half of the participants pointed to spirituality as a self-care practice for indirectly coping with moral distress. Participant No. 6 noted:

*“I think spirituality is a kind of self-care that I have to care of my health and well-being. When facing older adults in critical conditions, I have to relieve myself. Thinking and recalling of the most powerful nature helps me to tolerate these situations and continue to be a nurse. Without any exaggeration, being a nurse, especially in ICU, help me to be more spiritual than before. However, I am working in this unit for about 2 years.“*

Also, participants frequently used phrases and words that described calming, comfort, belief, peaceful, and mysterious ways. Here is the statement of participant No. 4:

*“I feel like I have a very strong spiritual sense. I feel like some of my patients can feel that just by me going in the room and calming them… I feel like I don’t have an issue with… end of life. So I kind of help families through that. And I think that they do feel that.“*

### Burden of care

Caring for significantly ill older-adult patients had some barriers that hampered the CCNs’ ability to provide the wanted care. Three subthemes pointed to the following:

Futile care was described by two nurses (participants No 7 and 8):

*“It has become very difficult to care for patients who I know will never get better and have no quality of life (i.e., our chronic vent patients).”*

*“I feel that the lack of quality of care and unnecessary treatments/futile care happens more often than it should.“*

Care that is perceived as futile contributes to the highest level of moral distress among the interviewees. Two main resources of futile care that lead to moral distress involved failure to relieve suffering or pain, and use of extensive medical resources on a patient who is unlikely to survive. It was mentioned by 3 CCNs who had worked in ICUs for over 3 years.

Positive and negative emotions associated with end-of-life care are labeled “emotional work.“ The difficulties of end-of-life care also influence the emotions of nurses when an older adult dies. The emotional attachment between nurses and older-adult patients enables them to provide emotional care, which is identified as an important factor for patients and their families. Actually, the provision of end-of-life care for patients was noted as satisfying, and it made nurses feel pleased with their work. However, it was emotionally draining. Participant No. 3 said:

*“I really feel happy when the patients and their families trust me and say, ‘We are sure that you do your best for us.’ These statements are really interesting at first, but when I think more about them, they make me to have more responsibility more than usual. It will be more as the patients experience uncomfortable conditions, and there is no hope for his or her relief. That really bothers me and think that, ‘Have I missed something?’”*.

Moral distress was found to result from the feelings of powerlessness the participants felt when they were unable to make ethical decisions. In these situations, they found that nurses perceived that “others” were in greater positions of power of decision-making than they were. One of the CCNs defined this subtheme as lack of respect for the nurse’s knowledge when caring for older adults, which contributed to moral distress.

### Relationship

Nurses who were interviewed believed that patients and their families are the central focus for the provision of nursing care. CCNs identified that building connections with the family of older-adult patients in ICUs is crucial to forming trust and working jointly in the best interests of the patient. They are also aware that they need the participation of the family—not just for legal matters, but also to learn more about the patient.

However, some situations trouble nurses. For example, participant No. 1 noted:

*“There are some situations which you know the patients better and want to care him/her better, … based on what you have learnt. However, the family insists on to not do it or do it in other way. You sit down and explain it to them, and unfortunately, it just goes right over their head. You try all you can to have them understand but they just—not that they don’t listen—they just don’t comprehend it.“*

*“I think here in intensive care, we have a lot of times where those patients who may have a very critical, chronic illness—that they’re… not going to get better, and they’re going to have no quality of life. But the family wants them to have invasive procedures and keep them on a ventilator and do all these things that are not… I think if the patient was able to, they probably would not have made the decision for themselves. But we have to do that a lot, and a lot of times, the family wants that. And you can have as many family meetings as you want. The family’s stringent about what they want.“*

CCNs cooperate with the patient and family, as well as the interdisciplinary team (IDT). Collaboration with members of the IDT has advantages and disadvantages. One disadvantage reported by participants is the experience of moral distress.

CCNs reported instances of moral distress associated with their professional relationships with nursing peers, as well as with other members of the healthcare team. The majority of the examples shared by CCNs in this study were associated with interactions with nurses and physicians. CCNs reported that conflicts with physicians occurred more often than issues that resulted from patients’ wishes not being honored. For example, participants No. 2, 4, and 5 stated, respectively, that:

*“There are commonly situations that MD’s do not respect the views of bedside nurses. These situations have affected me to reevaluate my role in the hospital.“*

*“It is morally distressing when patients are not receiving high-quality and beneficial care.”*

*“I had a situation when two doctors did not agree with my clinical assessment and dismissed my concerns when a patient’s change in status was noticed. I was not supported by my doctors and felt belittled and ignorant.”*

Participants reported their experiences with moral distress in the institutions where they were employed. Individual personalities and interpersonal interactions contribute to positive and negative experiences within the organization, which were also addressed by CCNs.

Participant No. 3 explained that she enjoyed the support of both her managers and nursing peers at a healthcare facility when she was hired as a new nursing graduate. She benefitted from a healthy workplace environment and had colleagues who “had your back.” Although she does not have the same support at her current healthcare facility, she had every expectation of a similar work environment, so she now appreciates how “spoiled” she was in her first job. She noted:

*“You see and you experience some pretty awful things on a day-to-day basis. So to not have a sense of teamwork, it would definitely make it hard to get through the 12-h shift.“*

*“Our nursing staff was very present in the medical rounds in the morning. He described the patients` conditions and let CCNs to share their medical information which they have learnt. Beside this, he involves the patients as well as his/her family to explain what he has experienced during his/her illnesses. He believed that ‘each patient is a book’ that can help nurses to learn more. You know, I think he was trying to limit the gap between theory and practice.“*

### Organizational issues

Lack of staff was problematic for end-of-life care in ICUs. Specifically, the amount of time the staff could spend with dying patients was reduced. This lack of time prevented the interviewees from providing the quality of care they felt they were capable of. Subsequently, they experienced distress when unpredicted deaths occurred, especially when older-adult patients died alone because of staffing issues.

For some patients whom death had been recognized, their family members may choose not to be present and this choice was incongruent with the staff’s priority to have the family present. This scenario can cause moral distress because the staff may think that “the right thing” is not being done. Participant No. 2 stated:

*“Because of lack of staffing was not due to lack of budget, but because no one wanted to work. Staffing got even worse when someone called in sick, and the administration was not able to get a replacement.“*

*“Sometimes, instead of one-hour lunch, you just do 30 minutes because you just want to finish it.”*

The next subtheme is inadequate training, preparedness, education, or mentoring. Nurses describing this theme valued competence and expertise. Furthermore, they believed that they were inadequate when they floated to another unit, were unfamiliar with an older-adult patient’s medical diagnosis, lacked skills required to care for patients, or did not have sufficient skills to operate unfamiliar equipment. Overall, the interviewees felt a duty to their patients. But at critical times, they were unable to act appropriately, due to a lack of training, preparedness, education, or guidance by another nurse.

As one nurse expressed, *“We need to be better advocates for ourselves.”* This sentiment included involving themselves in the creation of policies and procedures, and continually seeking training and support in order to develop expertise in unfamiliar areas. Participants No. 1, 5, 7, and 8 noted, respectively:

*"This was not what I was prepared to do.”*

*“Nursing school did not prepare me for this.”*

*“Some nurses are unable to talk to patients [or families] about [end-of-life decisions].”*

*"We aren’t prepared for that.”*

Participants in one-on-one interviews commented that a lack of support from the administration was evident when addressing staffing issues and dealing with the residents’ care plans. Participants felt unsupported and ignored by the administration, and they often felt trapped in their situations. They claimed the administration did not value the nurses’ input, made unilateral decisions, and ignored their suggestions about improving their work conditions. This was concerning for participants who felt that the administration would not provide the resources they needed. For example, participant No. 2 believed that:

*“When you have some residents who are really problematic, and you’re not getting the type of response you want… Not everyone in administration… I have to be honest with you. You can have a supervisor who is very concerned and into the problem and will jump into it. But you can get some who will keep on… pushing everything back on you to do it, despite the fact that you are up to your neck.”*

Moreover, participants No, 1, 4, 5, and 8 respectively stated:

*“My work performance is impacted when I feel like I am being pulled in many directions.”*

*“I feel like I have become less empathetic and careless, due to workload.”*

*“No helper available when I was busy. I felt so much stress and so behind in care.”*

*“I was not supported in situations that were comfortable. I feel that my coping and work performance became stressed, and I didn’t work as efficiently or effectively.”*

## Discussion

The intensive care unit is a unique working environment where nurses often deal with life threatening situations, and face frequent ethical dilemmas while providing care. In circumstances where patients are treated in the ICU for an extended period (e.g., due to terminal condition, advanced age, or multi-organ dysfunction), moral distress can be a common experience among healthcare professionals. Many factors have been identified to be positively correlated with high moral distress among ICU staff such as job dissatisfaction, high patient-healthcare ratio, and perceived stress from patient demands [38].

Moral distress among ICU staff has recently received more attention in the literature. Moral distress has been linked with quality of care, clinical decision making accuracy, work satisfaction, job retention, and patient satisfaction [38, 39]. Accordingly, various tools have been developed to assess and quantify the presence of moral distress and its related factors, as well as its clinical consequences [40]. In addition to quantitative exploration, further qualitative analyses are needed to gain a deeper understanding of moral distress from the perspective of ICU staff, which can guide policymakers to generate a comprehensive perspective in understanding moral distress and formulate reliable strategies.

This study has revealed some of the central concerns related to moral distress experienced by ICU nurses treating older adults over a long-term period in Iran.

*Advocating* is relatation to moral distress as there are often discrepancies among what patients need, what the family wants, and what is allowed or disallowed by regulations. Nurses are often caught in the middle of these conflicting situations and take responsibility for decisions and face potential consequences. The consequences include the patient’s clinical outcome (made better or worse), while personal consequences include negative judgments from others, possible administrative sanctions, or conflict with other staff. On the other hand, choosing not to advocate avoids problems that may occur, but can also lead to feelings of guilt, as the patient’s needs are not met. The grey areas of right and wrong ultimately contribute to moral distress among nurses in their daily duties. As recommended by Dunger et al. [41], nurses need to have sufficient understanding of ethical decision making principles to avoid the consequences of their clinical decisions.

*Defense mechanisms* encompass the way nurses personally deal with overcoming their experience of moral distress. They reflect internal processes of self-generated mechanisms to form a protective barrier for a potential psychological threat that may arise from moral distress. As a part of their duty to maintain the quality of care, ICU nurses need to be able to handle (and often set aside) moral distress to perform their work. This is made more difficult when the working environment is not supportive. As mentioned by most participants, talking to peers and practicing spirituality are the most common ways of dealing with moral distress. Peer support and socially-supportive organizations are an important part of ICU settings, and therefore need to be nurtured by healthcare leaders. As previously discussed by Borhani et al. [42], sufficient support from management (including the ethical commission) positively impacts nurses by improving job satisfaction and retention.

It is undeniable that, in some cases, nurses’ moral distress is influenced by the patients themselves. Patients’ conditions may be irreversible or terminal which brings a sense of helplessness to nurses and may lead to the belief that the only option for them is to improve the patient's quality of life while they are alive. It is also common for patients and their families to build a relationship, and become more emotionally connected with their treating nurses. This may either increase nurses’ work satisfaction because of their success in gaining trust from the patient and family, or make their work emotionally demanding as they become more committed or emotionally invested. These conditions may co-exist and can lead to “*care burden*” as found in the present study.

Individuals in the Human Resource Management workforce are in position to support nurses in ensuring appropriate ICU staffing and continuous professional development. As quoted by the participants, inadequate staffing, insufficient training, and poor preparedness, education, or mentoring increased nurses’ burden and distress. Borhani et al. [42] discussed the importance of adequate staffing on maintaining nurses’ ability to give patients appropriate quality of care. Additionally, staff shortage impairs nurses’ job satisfaction in ICU patient care.

## Conclusions

This qualitative study contributes to the limited knowledge and understanding of the challenges nurses face in the ICU from an Islamic lens. Given that nursing is a global profession, this study provides a new insight for non-Muslim nurses who practice in Islamic countries. It also offers possible implications for implementing supportive interventions to improve the wellbeing or nurses and reduce moral distress.

## Data Availability

The datasets used and/or analyzed during the current study are available from the corresponding author on reasonable request after approval from all the authors.

## Abbreviations

ICU: Intensive care unit
CCN: Critical care nurse
BSN: Bachelor of Science in Nursing
MSN: Master of Science in Nursing
IDT: Interdisciplinary team
